# Bibliography

**Published:** 1917-09

**Authors:** 


### BIBLIOGRAPHY

BRADY'S DENTAL METALLURGY. This new work on metallurgy by Edwin P. Brady, D.D.S., professor of chemistry, physics and metallurgy in Washington University Dental School is just issued by Lea and Febiger of Philadelphia. The text is designed to give to the dental student a knowledge of the physical properties of the metals he encounters in the course of instruction in college. Dr. Brady has had considerable experience in imparting instruction in the various lines of dental applied chemistry, and he has used good judgment in classifying the material for his book in a logical and pedagogic form. His idea of carrying this course concurrently with qualitative analysis is a good one, as it unquestionably adds interest to the qualitative course and assures more accurate knowledge of the technical treatment of metals in the metallurgic laboratory. The chapter on gold is well worth the price of the book, and the two chapters on analytical methods are very instructive. The book is well written and has the advantage of being compiled by a dental teacher of experience who makes his text interesting by constant reference to its technical relations. We believe it is one of the best texts on this subject available, and it should be of great value to the dental practitioner as well as the student.

